# Advances in the treatment of intraocular malignancies: A literature review

**DOI:** 10.3389/fmed.2022.975565

**Published:** 2022-10-18

**Authors:** Yanyan Cui, Shan Yin, Xuejiao Qin, Wanzhen Jiao, Anqi Ren, Fei Wang, Bojun Zhao

**Affiliations:** ^1^Shandong University of Traditional Chinese Medicine, Jinan, China; ^2^The Second Hospital of Shandong University, Jinan, China; ^3^Department of Ophthalmology, Shandong Provincial Hospital Affiliated to Shandong First Medical University, Jinan, China; ^4^Shengli Oilfield Central Hospital, Dongying, China

**Keywords:** intraocular tumors, malignant tumors, treatment, metastatic carcinoma, diagnose

## Abstract

Intraocular malignant tumors including primary and metastatic tumors, are mainly found in Retina and uvea, and very few cases originate from the sclera and optic nerve. Intraocular tumors can endanger the patient's vision and even life, and proper treatment is vital. There have been several traditional treatments for intraocular tumors, such as radiotherapy, chemotherapy and surgery. In recent years, new methods have been developed in clinical applications including anti-VEGF and gene therapy. This paper aims to provide a timely review about recent progress in the treatment of intraocular malignant tumor.

## Introduction

Intraocular malignant tumor is a rare disease in eyes but often seriously affects vision and even threatens life because of its location and growth characteristics. Once diagnosed, this tumor needs to be treated timely. Intraocular malignant tumors include primary malignant and metastatic tumors, and the most common sites of malignancies are uvea and retina. Choroidal melanoma and retinoblastoma are the most common primary intraocular malignant tumors in adults and children respectively (1). Choroidal metastatic carcinoma is the most common intraocular malignant tumor because of its abundant blood supply (2). The incidence of intraocular tumors is low. Suspicious intraocular malignant tumors need to be checked regularly every 1–3 months. If the patient's condition is stable, patients should be checked every 6 months. The follow-up times should be increased to protect the useful visual acuity of the patient if the tumor is close to the optic disc or macular fovea.

At present, many methods, such as radiotherapy, laser therapy, chemical therapy, surgical treatment, and anti-vascular endothelial growth factor (VEGF) injections, are available for the treatment of intraocular malignant tumors. The advantages, disadvantages, and indications of each treatment is discussed in following sections.

## Radiotherapy

Radiotherapy can be divided into proton beam, stereotactic, and short distance radiotherapy methods in accordance with the distance between the radiation source and the tumor (3). Radiotherapy is an effective treatment for malignant tumors after surgery and chemotherapy (2). At present, no report of stereotactic radiotherapy used to treat intraocular malignancies is available. Proton beam radiotherapy, also known as long-distance radiotherapy, includes X-rays, γ-rays, cobalt-60 (Co-60), and electron radiation (4). Proton beam radiation can cause cell damage or induce cell death by damaging the cell's DNA. When the radiation energy reaches the ionization absorption peak, the rapid death of cells may occur.

Proton beam radiation is used to treat malignancies because of its superior biophysical properties in term of dose deposition in tissues (5). Patient receiving proton beam radiation undergoes surgical placement of tantalum marker rings. These rings are placed at the tumor border on the sclera and serve as radiographic markers of the tumor edge for treatment planning and daily image guidance. After surgery, the patient receives radiotherapy, in which an immobilization device is prepared and the markers are imaged on X-ray to confirm their three-dimensional positioning in the eye (6). The damage to the surrounding tissues is minimal, and the damage to the optic disc and macula can be avoided during the proton beam radiation (7). Tumor should be carefully located before treatment, and the treatment should be separated into several times to increase the efficiency of radiation (4). This therapy is predominantly applied in the treatment of choroidal melanoma and cranial osteoma. Superficial tumors or tumors involving inner tissues are likely to absorb the energy, thus easily reaching the absorption peak and indicating suitable treatment (8). This treatment is not suitable for tumors involving the macular and optic disc. The vitreous hemorrhage and neovascular glaucoma after radiotherapy indicates the deterioration of the tumor. Wiegel et al. (9) reported 50 patients (65 eyes) with choroidal metastatic carcinoma treated with proton beam radiotherapy of 40 Gy. A total of 50 and 15 eyes are symptomatic and asymptomatic respectively, and the average followed time is 5.8 months (1–44 months). Among the 50 symptomatic eyes, 18 had improved visual acuity by at least two lines, 25 had unchanged visual acuity, and 7 had decreased visual acuity. The condition of 15 asymptomatic eyes was stable after treatment.

Plaque brachytherapy works through suturing a radioactive plaque temporarily to the episcleral to deliver a fixed dose directly to the tumor. The plaque is positioned appropriately to deliver the desired radiation dose to the entire tumor. The operative localization of the plaque placement is carefully guided by transillumination, ophthalmoscopic observation, or ultrasonography. The radioactive source adopted in the eye has been explored for a long time. In 1930, Moore first applied radon needle in the treatment of malignant choroidal soft tissue tumors[ref]. In 1939, Lommatsch adopted Ru-106 in the treatment of choroidal malignant tumors, and Sealyetal was the first to use I^125^ in the treatment of intraocular tumors. I^125^ has gradually replaced other radioactive sources and is now widely adopted in clinics due to the advantages of strong organizational permeability, dose standardization, and compact size (10–12). Other radioisotopes include cobalt-60 (Co-60) and palladium-103 (Pd-103). The theory of radiation application is to make a circular metal pad with radioactive sources on the inner surface of the sclera in accordance with the diameter of the sclera, implant it under the bulbar conjunctiva for 2–7 days, and then remove the applicator after the tumor tissue has absorbed a sufficient dose of radiation (13). The therapy can be used in combination with transpupillary thermotherapy (TTT) or with a radiation aid called a D-collimator to control the radiation range precisely (14). Shields et al. (15) used radiation application to treat 36 patients with choroidal metastatic cancer. Of these patients, 27 cases (75%) had radiation application as first-line therapy, whereas 9 cases (25%) had radiation application as second-line therapy after the failure of other treatments. In this study, average therapeutic doses of 68.80 and 235.64 Gy were adopted to irradiate the apex and base of the tumor, respectively. The average total treatment time was 86 h, after 3 months of treatment, the average mass thickness of 34 cases (94%) were halved, and the tumor disappeared at followed up of 11 months Chen et al. studied patients with choroidal melanoma and showed that the concentrations of VEGF-A and placental growth factor (PLGF) in the aqueous humor of patients with uveal melanoma (UM) increased after Iodine^125^ plaque therapy (16). Plaque brachytherapy is effective for the treatment of the tumors that involved in the optic nerve who had enucleation previously (17). This therapy is predominantly adopted for small tumors with evident growth tendency or medium-sized tumors but the patient still has a certain degree of vision (18).

Inconclusion, each subtype of the radiotherapy has its advantage and disadvantages, we should carefully consider their systematic condition and symptoms before making the choice.

## Laser therapy

The current laser treatments for eye tumors can be divided into argon laser photocoagulation, TTT, and PDT in accordance with the principles and mechanisms of action (19).

Argon laser photocoagulation can block the supporting blood vessels of tumor tissue and destroy tumor tissue by irradiating the tissue for 0.2–0.3 s with a wavelength of 532 nm and an average energy of 350 mW (20). The treatment is suitable for small tumors located at the posterior pole (20, 21) far from the optic disc. If the tumor is large, the argon laser photocoagulation is sometimes combined with cryotherapy. The common complications of photocoagulation are hemorrhage, macular injury, and secondary choroidal and retinal detachment (22, 23).

TTT was introduced for managing choroidal melanoma in 1994 and is found to have fewer complications, higher penetration, and higher tumor destruction rate compared with laser photocoagulation. TTT irradiates the target tissue through the dilated pupil by using the following parameters: infrared laser of wavelength, 810 nm; energy, 300–600 mW; and spot diameter, 1.2 mm. Destruction of cell membrane and portion with gradually increasing temperature (45–60 °C). This strategy leads to the change in the cell microenvironment and signal transduction pathway, resulting in cell death. Given that the infrared laser has a long wavelength and strong penetration, the laser can reach sub-retinal tissues. Due to its high melanin content, choroidal melanoma easily forms gray–white plaques when exposed to infrared laser. Thus, choroidal melanoma is sensitive to TTT treatment. At present, TTT is predominantly suitable for small retinoblastoma without sub-retinal metastasis, choroidal melanoma, high-risk melanoma with diameter smaller than 4 mm (24), and tumors close to the disc or macular area (20). Shields et al. (25) adopted TTT to treat 188 retinoblastomas (80 eyes of 58 cases), of which 161 tumors (85.6%)were in complete remission, and 27 (14.4%) had relapsed. Minimal treatment intensity is considered for retinoblastoma with diameter smaller than 3.0 mm to achieve satisfactory results. This method is also feasible and effective for posterior polar small choroidal metastatic carcinoma. Furthermore, TTT combined with Ru-106 radiotherapy can be adopted to treat medium-sized choroidal metastatic carcinoma (26). The common complications of TTT include macular pucker, macular edema, retinal vein occlusion, vitreous hemorrhage, subretinal hemorrhage, and neovascular glaucoma (27).

PDT is a form of laser therapy that targets abnormal capillaries and is useful for the treatment of intraocular neovascularization and neoplasms (28). PDT involves the intravenous administration of a photosensitizing chemical substance followed by the targeted application of a low-power and long-duration infrared laser beam (29). PDT irradiates the lesion with a laser of a specific wavelength, and the laser activates the photosensitizer that reaches the target tissue through the vein and produces some free radicals or highly reactive singlet oxygen, thus leading to cell lysis and death, with no damage to the normal tissue (30). At present, verteporfin is the most commonly used photosensitizer in ophthalmology. Verteporfin causes platelet aggregation and thrombosis after continuous irradiation by using a laser with a wavelength of 689 nm for 83 s, which blocks the blood vessels in the lesion area (19). This therapy has been adopted to treat a variety of intraocular tumors, including choroidal hemangioma, metastatic retinal tumor, angiogenic tumor, choroidal neovascularization secondary to choroidal osteoma, and retinal astrocytoma (31, 32). PDT acts through two mechanisms in intraocular tumors: (1) direct tumor destruction *via* selective cytotoxic activity against tumor cells, and (2) promotion of intraluminal photothrombosis in vessels supplying the tumor (33). In a study of 12 amelanotic or lightly pigmented small choroidal melanomas managed with PDT, Turkoglu et al. found complete tumor regression after 1 (*n* = 3, 25%), 2 (*n* = 3, 25%), and 3 (*n* = 2, 17%) sessions of primary PDT with stable or improved visual acuity (34). A study consisted of 40 eyes with 58 choroidal metastatic tumors treated with PDT, showed promising results, and achieved tumor control with 1 (*n* = 32 tumors [71%]) or 2 (*n* = 3 tumors [7%]) sessions. The study also showed that the primary cancer location or ocular tumor features (e.g., size, location, color, shape, related SRF) did not affect the tumor control rate (35). PDT is a well-tolerated outpatient modality for the treatment of selected benign or malignant intraocular tumors. Complications include sub-retinal exudate and exudative retinal detachment (36, 37). Extremely thick lesions may not be eligible for photodynamics therapy (PDT) because the 689 nm wavelength laser may not penetrate the entire tumor (38). Theoretically, PDT can be used together with systemic chemotherapy. Along with immunotherapy or hormone therapy, PDT is the preferred in the treatment of the patients with bilateral, multifocal choroidal metastases (38). Common complications include optic neuropathy, macular degeneration, cataract, and neovascular glaucoma.

## Chemotherapy (Chemical volume reduction)

Chemotherapy controls tumor growth by the local or systemic administration of chemotherapeutic drugs. Commonly used drugs include carboplatin, vincristine, cyclosporine, docosahexaenoic acid, and paclitaxel. Chemotherapy is predominantly adopted for tumors that occur binocularly and tumors with large volume that cannot be controlled by local treatment alone especially tumors that cause sub-retinal effusion and retinal detachment. Tumors with extraocular or systemic metastasis can be treated with combined therapy. The common side effects of this therapy are myelosuppression, local tissue necrosis, thrombophlebitis, and neurotoxicity (39). Studies confirmed that the control rate of retinoblastoma (Table 1: R-E groups I–IV) treated with chemotherapy alone can reach 51–65%. Furthermore, the control rate of chemotherapy combined with other treatments can reach 62–100%. For retinoblastoma (Table 1: R-E group V), the recurrence rate of chemotherapy alone is 63–75%. (40–43). The study indicates that retinoblastoma is more likely to respond to systemic chemotherapy if the lesions are located in the macula and if the patient is older than 2 months of age (40).

**Table 1:** Classification of retinoblastoma (Reese-Ellsworth).

**Group**	**Chances for treatment**	**Description**
Ia	Very favorable	Solitary tumor < 4dd;behind the equator
Ib		Multiple tumors < 4dd;behind the equator
IIa	Favorable	Solitary tumor 4-10dd;behind the equator
IIb		Multiple tumors 4-10dd;behind the equator
IIIa	Doubtful	Any lesion anterior to equator
IIIb		Solitary tumor>10dd;behind the equator
IVa	Unfavorable	Multiple tumors>10dd
IVb		Any lesion anterior to ora serrata
Va	Very unfavorable	Tumor involving >50%of the eye
Vb		Vitreous seeding

dd, disc diameters.

A three-agent combination (carboplatin, vincristine, and etoposide) is commonly used in intravenous chemotherapy (44). Sometimes, other agents, like topotecan or cisplatin, can be additionally administered through intravenous chemotherapy in accordance with the patient's response to agents (45).

Intra-arterial chemotherapy (IAC) in the treatment of intraocular tumors is advent recently (46). IAC involves the highly selective injection of 3–5 mg melphalan into the ophthalmic artery (47). This therapy is often used to reduce the size of the tumor and facilitate the local treatment of intraocular tumors, such as laser photocoagulation and TTT (20). Shields et al. demonstrated that IAC can be particularly successful at treating advanced tumors (48). Meel et al. (49) received the intra-arterial injection of chemotherapeutic drugs with a therapeutic dose of 20.1 ± 11.9 mGy per eye with a fluoroscopy time of 8.5 ± 4.6 min. Among the nine patients treated, 8 had improved or without change of visual acuity, thereby showing that IAC is an effective and safe treatment. In many centers, IAC has been widely adopted as the primary therapy for retinoblastoma, and numerous publications reported successful treatment outcomes (50). Severe local complications comprise phthisis of the affected eye, suprachoroidal hemorrhage, vitreous body hemorrhage, optic nerve palsy, and papillary edema (47).

The development of new materials also provides a new way for chemotherapy. The retrobulbar injection of carboplatin nanoparticles can previously be transported to the vitreous and retina through the sclera and can be continuously released for 72 h without evident side effect on the human body. The tissue penetrability of nanoparticles is high, which can promote the absorption and utilization of drugs, and IAC may be an effective adjuvant therapy for retinoblastoma with vitreous metastasis. However, the long-term pharmacological and clinical effects of IAC need to be further studied. Kalta et al. (51) divided six retinoblastoma patients into three groups who were about to receive monocular enucleation. Each patient has received 1 mL (10 mg·mL^−1^) nanoparticle carboplatin. Eyeballs are removed at 6, 24, and 72 h separately, and the drug concentrations in retina, vitreous, choroid, and lens were measured at the time the eyeball was removed. Results showed that the highest drug concentration in retina was detected 24 h after injection. The concentration of drug in the vitreous decreased from 2.17 ± 0.86 mg·g^−1^ at 6 h after injection to 0.39 ± 0.11 mg·g^−1^ at 72 h after injection. The trace drug was detected in the choroid and lens 6 h after injection and almost disappeared at 24 h after injection. High drug concentration was obtained in the vitreous and retina, thereby making IAC an effective treatment for retinoblastoma with vitreous metastasis.

### Surgical excision

Surgical excision can be divided into the local enucleation of tumor and the enucleation of eyeball. The approaches for local tumor resection include transretinal and trans-scleral approaches. Transretinal tumor excision is mostly adopted for the treatment of tumors located in the posterior pole. In addition, the trans-scleral tumor removal is mostly adopted for the treatment of tumors located in the ciliary body and its periphery. Trans-scleral tumor removal is difficult and has not been widely carried out in China (30). The local resection of tumor should have a strict indications, and most patients treated by local resection need have further adjuvant therapy, such as photocoagulation and radiotherapy. The eyeball enucleation is adopted to treat intraocular malignant tumors with large volume, highly progressive involvement of the optic disc, secondary high intraocular pressure, retinal detachment, and unrecoverable visual acuity. Simple enucleation has disadvantage in improving the quality of life of patients and may increase the distant metastasis of the tumor. Thus, the eyeball enucleation is not recommended (52). Epstein et al. (53) conducted a retrospective analysis of 324 consecutive patients with retinoblastoma treated in the Oncology Department of Wills Eye Hospital and found that the proportions of enucleation of monocular retinoblastoma were 96% in 1974–1978, 86% in 1979–1978, and 75% in 1984–1988. In addition, the eyeball enucleation rate in patients with binocular retinoblastoma shows a downward trend.

### Clinical cryotherapy

Clinical cryotherapy achieves a therapeutic effect through the rapid lowering of the temperature of the tumor tissue to −90 °C, formation of ice crystals, protein denaturation, pH changes, and the destruction of vascular endothelial cells that lead to the ischemic death of tumor tissues (54, 55). Clinical cryotherapy is used to treat small tumors in front of the equator and is the first choice for sub-retinal metastatic tumors near the serrated margin. This therapy includes three courses of treatment with an interval of 1 month. Tumors with diameter larger than 3.5 mm, thickness greater than 2.0 mm, and located behind the equator or tumors with vitreous metastasis are not suitable for this therapy. The main side effects of this therapy are eyelid edema and transient retinal detachment (54, 55).

### Intraocular injection of anti-VEGF treatment

VEGF is considered an important factor to promote pathological or physiological angiogenesis (56, 57). VEGF can promote tumor angiogenesis and change vascular permeability. In addition, VEGF can regulate important signal pathways related to tumorigenesis, including the function of tumor stem cells and the origin of tumor cells (56). VEGF-A is a key proangiogenic factor associated with angiogenesis in numerous tumors (58). Similar to those in previous studies, Missotten has detected abnormally high intraocular concentration of VEGF-A in eyes with UM (59). Increased serum VEGF is also detected in patients with metastatic UM (60). Anti-VEGF treatment is currently used for the treatment of intraocular tumors.

Bevacizumab is a recombinant humanized anti-VEGF monoclonal antibody containing 93% human gene framework and 7% mouse protein sequence. It can bind to all subtypes of VEGF and effectively inhibit neovascularization and tumor cell proliferation, metastasis, and spread (61). At present, FDA in the United States has approved the intravenous bevacizumab combined with chemotherapy in the treatment of metastatic colorectal cancer, non-small cell lung cancer, recurrent pleomorphic glioblastoma, and metastatic breast cancer M471 (62, 63). The anti-VEGF drugs adopted for intravitreal injection are Ranibizumab, Conbercept, and Aflibercept. They are currently adopted for the treatment of age-related macular degeneration, retinal vein occlusion, and diabetic retinopathy. Ranibizumab, a recombinant humanized anti-VEGF monoclonal antibody fragment, has stronger penetrability, shorter half-life, and less clinical side effects than bevacizumab. Li et al. (64) cultured malignant melanoma cells and retinal pigment epithelial cells and stimulated by VEGF and ranibizumab and monitored the changes in cells by using a real-time cell electronic sensor. The proliferation ability of malignant melanoma cells increased by 40%, and the response of retinal pigment epithelial cells was not evident when given VEGF. Ranibizumab decreased the proliferation ability of malignant melanoma cells by 57.5%. Besides, the pigment epithelial cells decreased only slightly, indicating that tumor cells are sensitive to anti-VEGF therapy.

Amselem et al. (65) performed the vitreous injection of 4 mg bevacizumab into the vitreous body of a patient with choroidal metastatic carcinoma and bone and lung metastasis with the primary lesion located in the breast and revealed that the best corrected visual acuity was improved from 20/100 to 20/60. The B-ultrasound examination showed that the volume of the mass was reduced by half. Mason et al. (66) retrospectively studied 10 patients with choroidal melanoma who had received only a single intravitreal injection of bevacizumab. Six weeks later, the average visual acuity was improved from 20/100 to 20/86, and at the fourth month after injection, the average visual acuity was 20/95. Maudgil et al. (67) administered the intravitreal injection of bevacizumab to five patients with choroidal metastatic carcinoma, the deterioration was observed in four patients. Lin (68) thought the main reason was that exudation could limit the potential efficacy of intravitreal bevacizumab in this condition, as most choroidal metastases were associated with significant exudation. A study showed that bevacizumab significantly reduces the level of VEGF in the culture media from human UM cells, mouse melanoma cells, and co-cultured cells. Bevacizumab also inhibits cell tube formation and decreases the *in vitro* invasion of tumor cells (69). Rumana N reviewed seven patients with high-risk ocular melanoma, treated the patients with ranibizumab, and showed the role of intravitreal anti-VEGF for the treatment of the sequelae of local radiotherapy such as radiation retinopathy. Thus, these agents may be used as adjuncts in the treatment of UM (70). Fifteen reported choroidal was found by search “bevacizumab and choroidal metastasis” in pubmed and medline has been studied with different primaries. Fifteen metastases were treated with bevacizumab one or more times, and the treatment outcomes are listed in Table 2. The therapeutic effect of anti-VEGF in ocular tumors should be verified clinically. Precautions for anti-VEGF injection include antibiotic eye drops and strict aseptic operation. The potential systemic effects of anti-VEGF treatment include the development of thromboembolic events, systemic arterial blood pressure being raised, ventricular dilatation, contractile dysfunction.

**Table 2:** Choroidal metastasis treated by Bevacizumab.

**Study**	**Nb of patient**	**VA before treatment**	**VA after treatment**	**Primary cancer**
Aydin, Rukiye (71)	1	20/50	20/20	Cholangiocarcinoma
Margaret Wong (72)	1	20/80	20/30	Renal carcinoma
A Maudgil (67)	4-1	20/200	HM	Pancreatic cancer
A Maudgil (67)	4-2	20/60	20/120	Lung cancer
A Maudgil (67)	4-3	20/60	20/200	Breast cancer
A Maudgil (67)	4-4	*CF*	HM	Pancreatic cancer
L Amselem (65)	1	20/100	20/60	Breast cancer
V Fenicia (73)	3-1	20/50	20/20	Breast cancer
V Fenicia (73)	3-2	CF	20/25	Lung cancer
V Fenicia (73)	3-3	20/25	20/20	Breast cancer
Augustine (74)	1	20/125	20/20	Breast cancer
Kuo (75)	1	HM	HM	Not mentioned
Mansour (76)	1	20/200	20/40	Not mentioned
Wu (77)	1	20/100	20/60	Lung cancer
Yao (78)	1	CF	20/30	Breast cancer
C-J Lin (68)	1	20/200	20/400	Colon cancer
de la Barquera Cordero AS (79)	1	20/40	20/20	Lung cancer

## Gene therapy

Cancer is a series of diseases caused by acquired genetic abnormalities. A functional or therapeutic gene can be inserted to replace the defective endogenous gene and use oligonucleotides to reduce the products of defective genes in accordance with the type of gene mutation (80). Gene therapy is first applied in the treatment of rare or congenital diseases, such as primary immunodeficiency syndrome. At present, nearly 40 patients with adenine nucleosidase deficiency have been successfully treated with gene therapy. These cases have promoted the breakthrough of gene therapy in tumor (81). The application of gene therapy in the eye is still limited. O'reilly et al. (82) adopted RPE65 gene to treat the patients with congenital melanosis, and the patients' visual acuity has been improved. Moreover, visual field examination revealed that patients were more sensitive to small and short-term stimulation after treatment, suggesting the effectiveness of gene therapy. Recently, UM is found to have 15 gene phenotypes, thereby providing a theoretical basis for gene diagnosis and treatment of UM (83). Yang J showed that microRNA-145 (miR-145) played an important role in the development of UM, demonstrated that the levels of neuroblastoma RAS viral oncogene homolog (N-RAS) and VEGF in UM tissues were elevated, and revealed N-RAS and VEGF as downstream targets of miR-145 (84). José M et.al conducted a study about uveal melanoma (UM), and built a novel ferroptosis-related seven-gene signature (ALOX12, CD44, MAP1LC3C, STEAP3, HMOX1, ITGA6, and AIFM2/FSP1). They demonstrated that it could accurately predict UM prognosis and was related to Mast cells resting, which provides the potential for personalized outcome prediction and the development of new therapies in the UM population (71). With the rapid development of the science and technology, more and more gene therapy will be researched and will be applied in clinical in the near future.

## Conclusion

The purpose of the treatment for intraocular malignant tumors is to preserve useful vision as much as possible. Small or suspected malignant tumors can be followed up regularly. TTT or local radiation outside the sclera combined with TTT is feasible to be used if the tumor shows an evident growth tendency. Local resection is useful for malignant tumors around the eyeball. Chemotherapy and radiotherapy are suitable for most intraocular tumors with satisfactory results. Anti-VEGF intravitreal injection is a simple and effective method with minimal side effects to patients and still needs further clinical observation. Comprehensively consideration of the patient's condition is recommended and an optimal treatment can then be selected, to low the recurrence and adverse reactions, in order to effectively improve the patient's vision and quality of life.

## Author contributions

YC wrote the manuscript. SY and XQ checked the manuscript. AR and FW edited the references. BZ checked the manuscript, tables, and references. All authors contributed to the article and approved the submitted version.

## Funding

This work was supported by the Shandong Nature Science Foundation of BZ (ZR2019MH111).

## Conflict of interest

The authors declare that the research was conducted in the absence of any commercial or financial relationships that could be construed as a potential conflict of interest.

## Publisher's note

All claims expressed in this article are solely those of the authors and do not necessarily represent those of their affiliated organizations, or those of the publisher, the editors and the reviewers. Any product that may be evaluated in this article, or claim that may be made by its manufacturer, is not guaranteed or endorsed by the publisher.
